# Interindividual variability in immune response to AAV ocular gene delivery across species impedes immunomonitoring

**DOI:** 10.1172/jci.insight.199587

**Published:** 2026-02-17

**Authors:** Duohao Ren, Gaelle A. Chauveau, Julie Vendomele, Emilie Cabon, Audrey Pineiro, Catherine Vignal-Clermont, Hanadi Saliba, Giuseppe Ronzitti, Anne Galy, Deniz Dalkara, Juliette Pulman, Divya Ail, Sylvain Fisson

**Affiliations:** 1 Université Paris-Saclay, Univ Évry, Inserm, Genethon, Integrare research unit UMR_S951, Evry, France.; 2 Genethon, Evry, France.; 3 Sorbonne Université, CNRS, Inserm, Institut de la Vision, Paris, France.; 4 Department of Neuro Ophthalmology and Emergencies, Rothschild Foundation Hospital, Paris, France.; 5 Centre Hospitalier National d’Ophtalmologie des Quinze Vingts, Paris, France.

**Keywords:** Immunology, Inflammation, Ophthalmology, Adaptive immunity, Biomarkers, Gene therapy

## Abstract

Adeno-associated viruses (AAVs) have been used in gene therapy, especially for inherited retinal diseases. Despite their effectiveness in gene transduction, immune responses to the AAV capsid and transgene products have been reported, which can compromise both the efficacy and the safety of AAV-mediated therapies. The eye is regarded as an immune-privileged organ where immune activity is constitutively suppressed. Here, we highlight that immunomonitoring in an ocular gene transfer reveals variable immune responses, whatever the species (human clinical trial, nonhuman primates, mice), the site of injection, the cassette, and the dose. We further explored factors contributing to this variability, investigating the potential correlation among immune parameters in a controlled experimental setting. In a syngeneic murine model after a subretinal injection of AAV, our results highlight an interindividual variability of immune parameters, emphasizing the importance of considering inherent variability among individuals when designing personalized therapies.

## Abstract 2

Gene therapy viruses can trigger unpredictable immune responses, which vary between individuals and may affect safety and effectiveness of retinal treatments. <div> <div> <div> </div> </div> </div>

## Introduction

Adeno-associated virus (AAV) vectors have become one of the most promising tools in gene therapy, largely because of their ability to deliver genetic material efficiently and safely into a variety of cell types. Unlike other viral vectors, AAV is nonpathogenic and exhibits minimal toxicity, making it suitable for treating genetic disorders such as hemophilia, muscular dystrophy, and retinal diseases (1–4). Since the first attempt of gene transfer in the retina with AAV in 1996, proving the efficiency of transduction (5), preclinical research in the application of AAV-mediated gene therapy for retinal diseases has been on the rise. In 2007, the first clinical trial using AAV to treat Leber congenital amaurosis was initiated (6), which eventually led to the first FDA-approved AAV-based ocular gene therapy, Luxturna (voretigene neparvovec), approved in 2017 for RPE65 mutation–associated retinal dystrophy (7). By the end of 2024, 142 clinical trials had been conducted or were ongoing targeting 12 retinal diseases with 6 AAV serotypes (ClinicalTrials.gov, keywords: AAV, retina). These outputs have provided convincing proof for the application of AAV-based gene therapies to treat retinal genetic diseases. However, an often overlooked or underreported aspect of AAV-based therapies, especially in the ocular field, is the immune responses induced by the AAV capsid and transgene product. The therapeutic success of AAV-based gene therapy is substantially influenced by the host’s immune response, which can on one hand limit the effectiveness of the treatment (8), and on the other cause adverse secondary effects such as inflammation.

Although the eye is considered as an immune-privileged organ (9), safety of ocular gene therapy mediated by AAV is not guaranteed. Indeed, studies have shown that microglial cells can be activated after subretinal injection of AAVs in murine models (10). Systemic humoral and cellular immune responses can also be elicited by AAV and transgene products that are delivered by subretinal injections (11, 12). A study evaluating the immune responses to AAV delivery in the retina of nonhuman primates (NHP) reported elevated levels of anti-AAV antibodies in the serum (systemic immune response) as well as ocular inflammation (local immune response) (11, 13). A study that compared immune responses to intravitreally delivered full (capsid plus genome) or empty (only capsids) AAVs in NHPs showed that the empty capsids triggered a lower immune response compared with full capsids. It also reported that all ratios of full or empty capsids resulted in production of neutralizing antibodies in the serum (14). Another study, evaluating the cellular immune response by immunostaining in NHPs after AAV subretinal injections in the retina, reported CD8^+^ T cell infiltration (15).

Immune responses have been observed and reported not only in animal models used in gene therapy research, but also in clinical trials. Ocular inflammation was reported in patients undergoing ocular AAV gene therapy, and both anti-capsid humoral and cellular immune responses were reported, indicating the potential strong side effects of the AAV gene therapy (16, 17). Often, patients in these trials are provided with immunosuppression regimens that aim at managing the immune response–related side effects (18, 19). Despite this, some patients developed ocular inflammation and systemic immune responses (20, 21). An intriguing point is that nonconsistent immune responses were observed whatever the condition in clinical trials. For example, in clinical trial NCT00749957, 3 of 12 patients developed ocular inflammation and 5 of 12 patients developed anti-capsid antibodies, while no patients developed anti-capsid T cell response (22). This has been supposedly attributed to differences in disease stage, treatment prior to gene therapy, genetics (23, 24), lifestyle, and environment (25), which are confounding factors that can influence the correlations among the immunomonitoring parameters in the patients and the efficiency of the gene therapy. Thus, it would be useful to test these findings in controlled animal models. Moreover, it would be additionally valuable to demonstrate that the same kind of immune response variability can be found whatever the species (human clinical trial, nonhuman primates, mice), the site of injection, the cassette, and the dose.

In the present study, we first highlighted the interindividual differences in the anti-AAV immune responses in an ocular gene therapy clinical trial and in an NHP experiment. Next, we conducted analyses of local and systemic immune responses induced by AAV injection, maintaining consistent biological and experimental parameters to identify potential correlations among immune parameters. To that end, we administered AAV vectors through subretinal delivery in syngeneic mice and evaluated various immune parameters, including antibody production, T cell response, local inflammation, and cytokine secretion. Despite the controlled conditions, we observed marked interindividual variability in immune responses, with only limited correlations identified among the assessed parameters.

## Results

### Interindividual variability of immune response is observed in the human clinical trial and NHP model after intravitreal AAV2 gene transfer.

**Figure 1:**
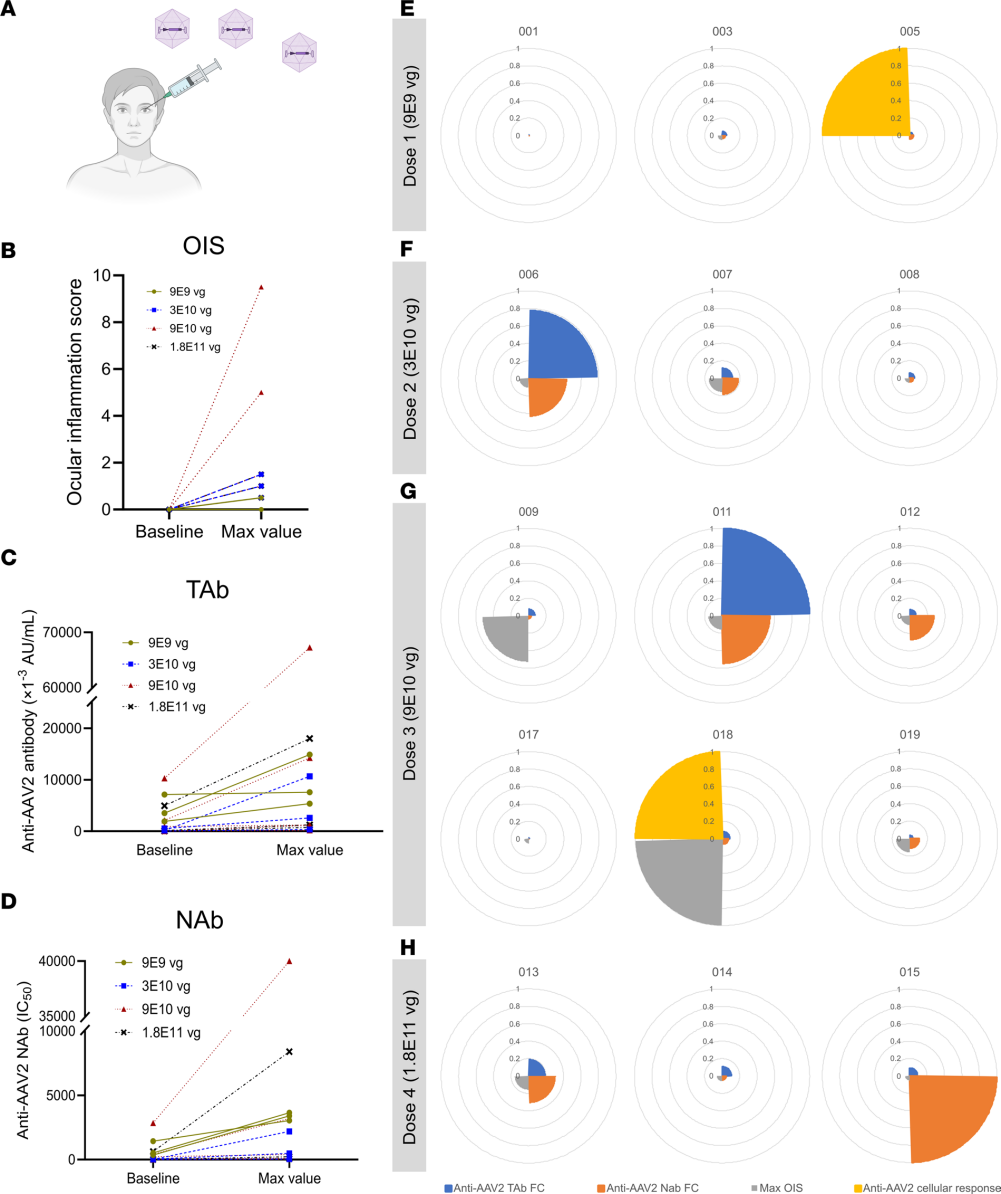
Interindividual variability of immune response is observed in the human clinical trial (NCT02064569) after intravitreal AAV2 gene transfer. (**A**) Schematic of ocular gene injections in patients. (**B**) Ocular inflammation score (OIS) in human patients who received intravitreal AAV injections at baseline and maximal value (obtained within 20 weeks after injection, except patient 003 for 40 weeks). (**C** and **D**) Total antibody (TAb) (**C**) and neutralizing antibody (NAb) (**D**) levels measured by ELISA and NAb assay against AAV2 in human patients who received intravitreal AAV injections at baseline and maximal value (obtained within 20 weeks after injection). IC_50_, half-maximal inhibitory concentration. (**E**–**H**) Immune profiles of individual patients, who received dose 1 (9 × 10^9^ vector genomes [vg]) (**E**), dose 2 (3 × 10^10^ vg) (**F**), dose 3 (9 × 10^10^ vg) (**G**), or dose 4 (1.8 × 10^11^ vg) (**H**) of AAV2, showing maximum ocular OIS, fold change (FC) of anti-AAV2 TAb and NAb, and anti-AAV2 cellular immune response. Each slice of the pie corresponds to one immune parameter, which is normalized against the highest value for the parameter. The code of each patient is shown above each chart.

Immunomonitoring after intravitreal AAV2 gene transfer showed immune responses in both a human clinical trial (26) and NHPs (11). Since different strategies yielded distinct outcomes, we investigated the potential relations between the different immunomonitoring data from a human clinical trial described previously (NCT02064569) (26) (Figure 1A). In this clinical trial, 15 patients diagnosed with ND4 Leber hereditary optic neuropathy were distributed into 4 cohorts and treated with an intravitreal injection of a recombinant AAV2 vector carrying the ND4 gene at 4 different doses. A composite global ocular inflammation score (OIS) was determined using 4 separate grades according to Standardization of Uveitis Nomenclature (SUN) classification (Figure 1B). In addition, total antibody (TAb) and neutralizing antibody (NAb) levels against the capsid were also measured by ELISA and NAb assays, respectively, in patients. TAb and NAb levels were generally increased in patients’ post-injection serum samples, with no clear dose effect (Figure 1, C and D). Immune profiles were generated for each patient, incorporating fold change of TAb and NAb against AAV2, maximal OIS, and anti-capsid cellular immune responses measured by IFNγ ELISpot in peripheral blood mononuclear cells (PBMCs) isolated from patients. The immunomonitoring revealed distinct patterns in patients who received the same dose, exhibiting different levels of OIS and cellular and humoral responses regardless of the AAV2 dose received (Figure 1, E–H, and Supplemental Table 1; supplemental material available online with this article; https://doi.org/10.1172/jci.insight.199587DS1).

**Figure 2:**
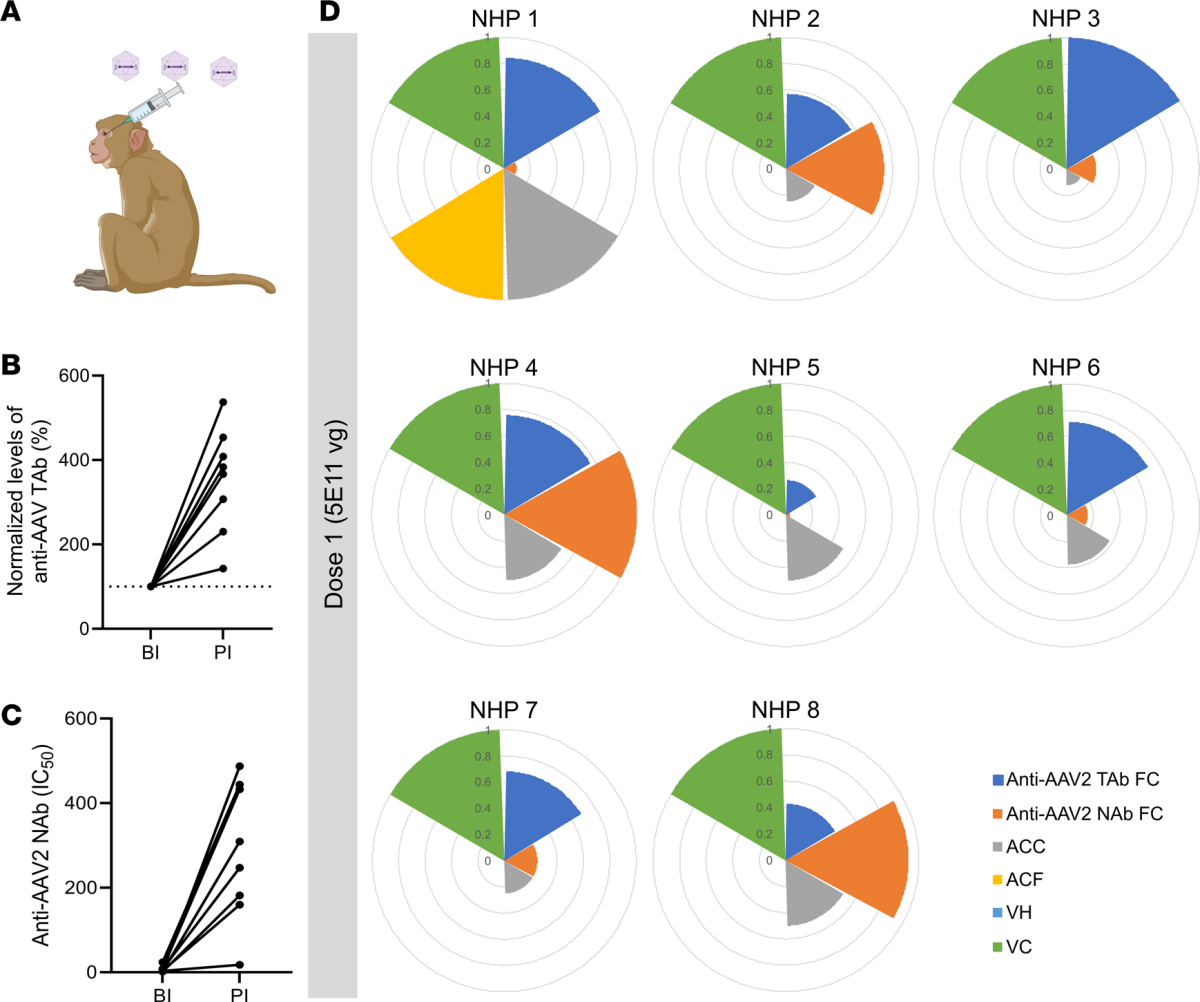
Interindividual variability of immune response is observed in the NHP model after intravitreal AAV2.7m8 gene transfer. (**A**) Schematic of ocular gene injections in NHPs. (**B** and **C**) Total antibody (TAb) (**B**) and neutralizing antibody (NAb) (**C**) levels measured by ELISA and NAb assay against AAV2 in NHPs that received intravitreal AAV injections with dose 1 (5 × 10^11^ vg) before injection (BI) and 2–3 months post-injection (PI). IC_50_, half-maximal inhibitory concentration. (**D**) Immune profiles of individual NHPs showing fold change (FC) of anti-AAV2 TAb and NAb, grading score at month 1 for anterior chamber cells (ACC), anterior chamber flare (ACF), vitreous haze (VH), and vitreous cells (VC). Each slice of the pie corresponds to one immune parameter, which is normalized against the highest value for the parameter. The code of each NHP is shown above each chart.

In order to further investigate immune responses under more controlled environmental conditions, immunomonitoring data from a study on NHPs were analyzed (11). All 8 NHPs received an intravitreal injection of 5 × 10^11^ vector genomes (vg) of AAV2.7m8 encoding ChrimsonR in both eyes (Figure 2A). Ocular inflammation was assessed by slit lamp 1 month after injection, and scores were determined by SUN classification on anterior chamber cells (ACCs), anterior chamber flare (ACF), vitreous haze (VH), and vitreous cells (VCs). ELISA and NAb assays were used to measure capsid-specific TAb and NAb levels 2–3 months after injection. Both TAb and NAb against capsid in NHP sera increased after AAV2 administration (Figure 2, B and C). Normalized immune profiles of NHPs highlighted varying contributions of humoral immunity and inflammation by slit lamp, including ACCs, ACF, VH, and VCs, to the overall response (Figure 2D and Supplemental Table 2). Both species exhibited variability in immunomonitoring outputs following intravitreal AAV2 injection.

### Systemic adaptive immune responses against both capsid and transgene product are induced after subretinal injections of AAV8-GFP-HY in mice.

**Figure 3:**
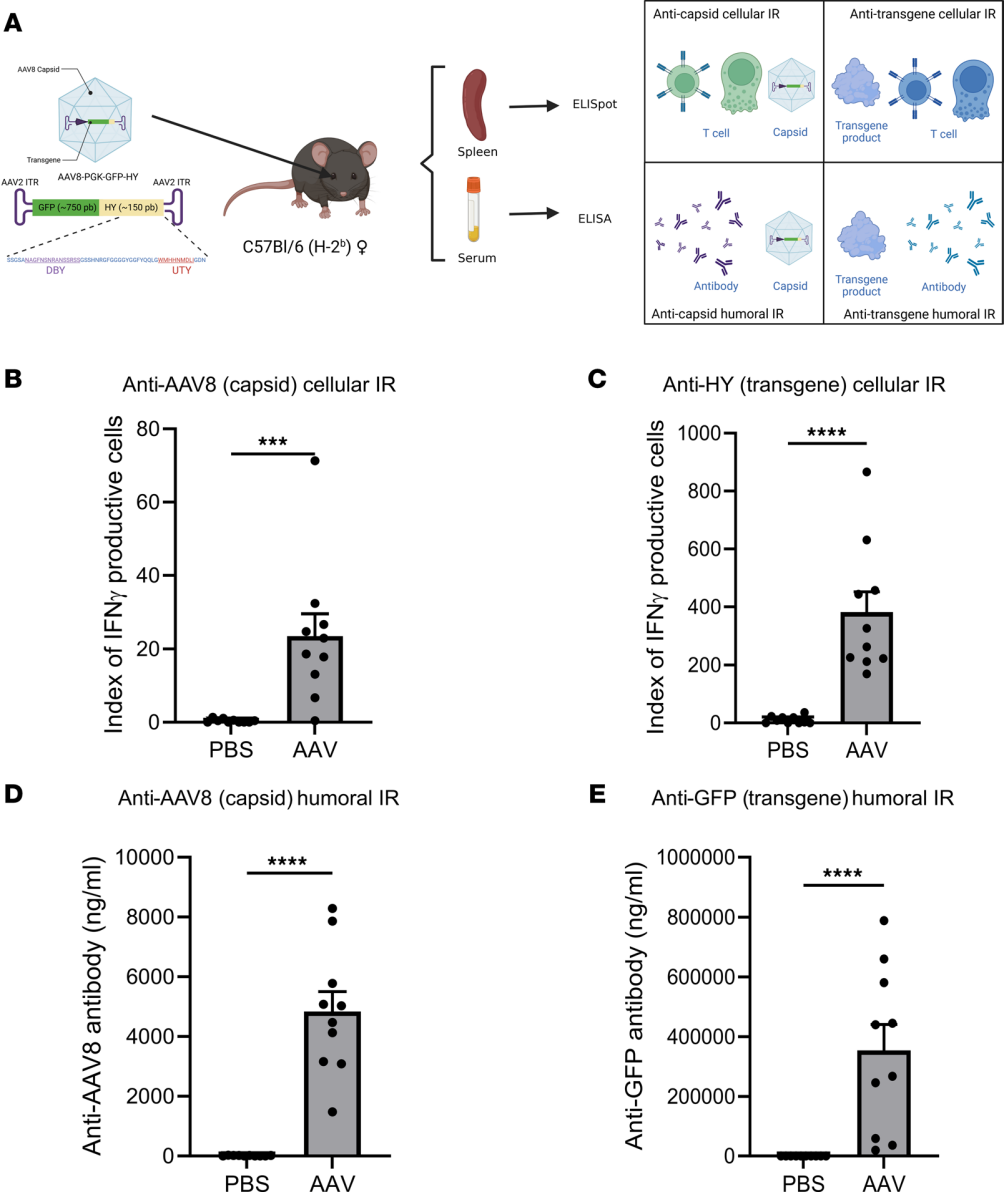
Systemic adaptive immune responses are induced after subretinal injections of AAV8-GFP-HY in mice. (**A**) Schematic representation of the experimental procedure showing the subretinal injection of AAV8-GFP-HY (5 × 10^10^ vg) in mice, followed by harvest of the spleen and serum 21 days after injection to test cellular and humoral responses against the capsid and transgene. (**B** and **C**) T cell activation measured by ELISpot assay against the AAV8 capsid (**B**) and the transgene-HY (**C**). (**D** and **E**) Antibody levels measured by ELISA against the AAV8 capsid (**D**) and the transgene-GFP (**E**). Results obtained from 2 independent experiments (*n* = 10 per group). Bars correspond to mean + SEM. ***P < 0.001, ****P < 0.0001 with unpaired Mann-Whitney test.

**Figure 4:**
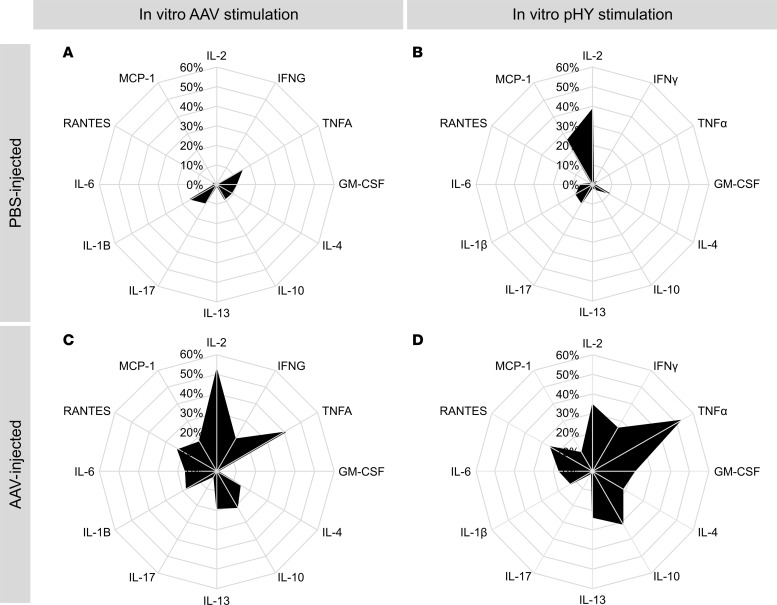
Global view of the systemic proinflammatory cytokine profile in mice 21 days after injection of AAV8-GFP-HY. (**A** and **B**) Cytokine profiles of PBS-injected group with AAV in vitro stimulation (**A**) and peptide HY (pHY) stimulation (**B**). (**C** and **D**) Cytokine profiles of AAV-injected group with AAV in vitro stimulation (**C**) and pHY stimulation (**D**). The 100% radar scale fits to the maximum value of cytokine secretion, and the black area values correspond to the mean of these cytokine secretions. Cytokines tested were interleukins (ILs); tumor necrosis factor-α (TNFA); interferon-γ (IFNG); granulocyte-macrophage colony-stimulating factor (GM-CSF); regulated upon activation, normal T cell expressed and secreted (RANTES; also known as CCL5); and monocyte chemoattractant protein 1 (MCP-1; also known as CCL2). The concentration of each cytokine in each condition can be found in Supplemental Figures 2 and 3 and Supplemental Table 3.

Because of the diversity in the disease stage and genome and environmental factors in humans and NHPs, syngeneic murine models seem to be pertinent to explore the consistency of the immune responses following AAV-mediated gene transfer. Previous studies have demonstrated that AAV subretinal injections can induce both humoral and cellular immune responses against capsids and transgene products that can be detected systemically in blood or in lymphoid organs in murine models (27–30); however, these studies typically focused on limited immune parameters like antibody production and local inflammation. To comprehensively assess the humoral and cellular immune responses against both capsids and transgene products, a male peptide named HY, which was known to induce systemic T cell responses (12, 31) in female mice, was packaged into AAV8 capsid along with GFP. The AAV8 vectors (5 × 10^10^ vg/eye) were administered into the eye of female C57BL/6 mice via subretinal injection (Figure 3A). Sera and spleens were collected 21 days after injection as previously described (12, 31) to analyze the humoral and cellular immune responses against the capsid and transgene product (Figure 3A). IFNγ ELISpot revealed a significant increase in activated T cells against AAV8 (*P* = 0.0001) and transgene product (*P* < 0.0001) in AAV8-injected mice compared with PBS-injected control mice (Figure 3, B and C, and Supplemental Figure 1A). HY peptides (pHY) are composed of DBY, activating CD4^+^ T cells, and UTY, activating CD8^+^ T cells (32). ELISpot assay showed an IFNγ secretion by CD4^+^ and CD8^+^ T cells specific to transgene product (Supplemental Figure 1, B and C). ELISA measurements showed that anti-AAV8 and anti-GFP antibodies significantly increased (*P* < 0.0001) after AAV8 subretinal injection in comparison with PBS-injected control mice (Figure 3, D and E). The systemic cytokine profile contributing to the inflammation and immune response in mice was evaluated following AAV8 subretinal injection. Spleen cells were isolated from mice that received either PBS or AAV8. These spleen cells were then stimulated in vitro with pHY or AAV8. A cytometric bead array was used to measure cytokines in the culture supernatant to determine the cell polarizations: Th1/Tc1 (IL-2, IFNγ, TNFα, GM-CSF), Th2/Tc2 (IL-4, IL-10, IL-13), Th17/Tc17 (IL-17), and those involved in inflammation and migration (IL-1β, IL-6, RANTES, MCP-1). Radar charts were generated to visualize the proportional production of these cytokines. Spleen cells from PBS-injected control mice mainly did not secrete cytokines (Figure 4, A and B). In contrast, spleen cells from AAV8-injected mice produced multiple cytokines (Figure 4, C and D). Among cytokines involved in inflammation and migration, only RANTES (*P* = 0.01) showed a significant increase (Supplemental Figure 2A). The Th1/Tc1 cytokines, IL-2 (*P* = 0.0046), IFNγ (*P* = 0.0001), and TNFα (*P* = 0.0388), were significantly upregulated (Supplemental Figure 2B) in response to in vitro AAV8 stimulation. Other cytokines remained unchanged (Supplemental Figure 2, A–D). Upon in vitro stimulation with pHY, cytokines such as RANTES (*P* < 0.0001), IFNγ (*P* = 0.0001), TNFα (*P* = 0.0007), and IL-10 (*P* = 0.0024) were significantly upregulated (Supplemental Figure 3, A–D).

### Local transgene expression in the retina is correlated with cytotoxicity against transgene-expressing cells after injection of AAV8-GFP-HY.

**Figure 5:**
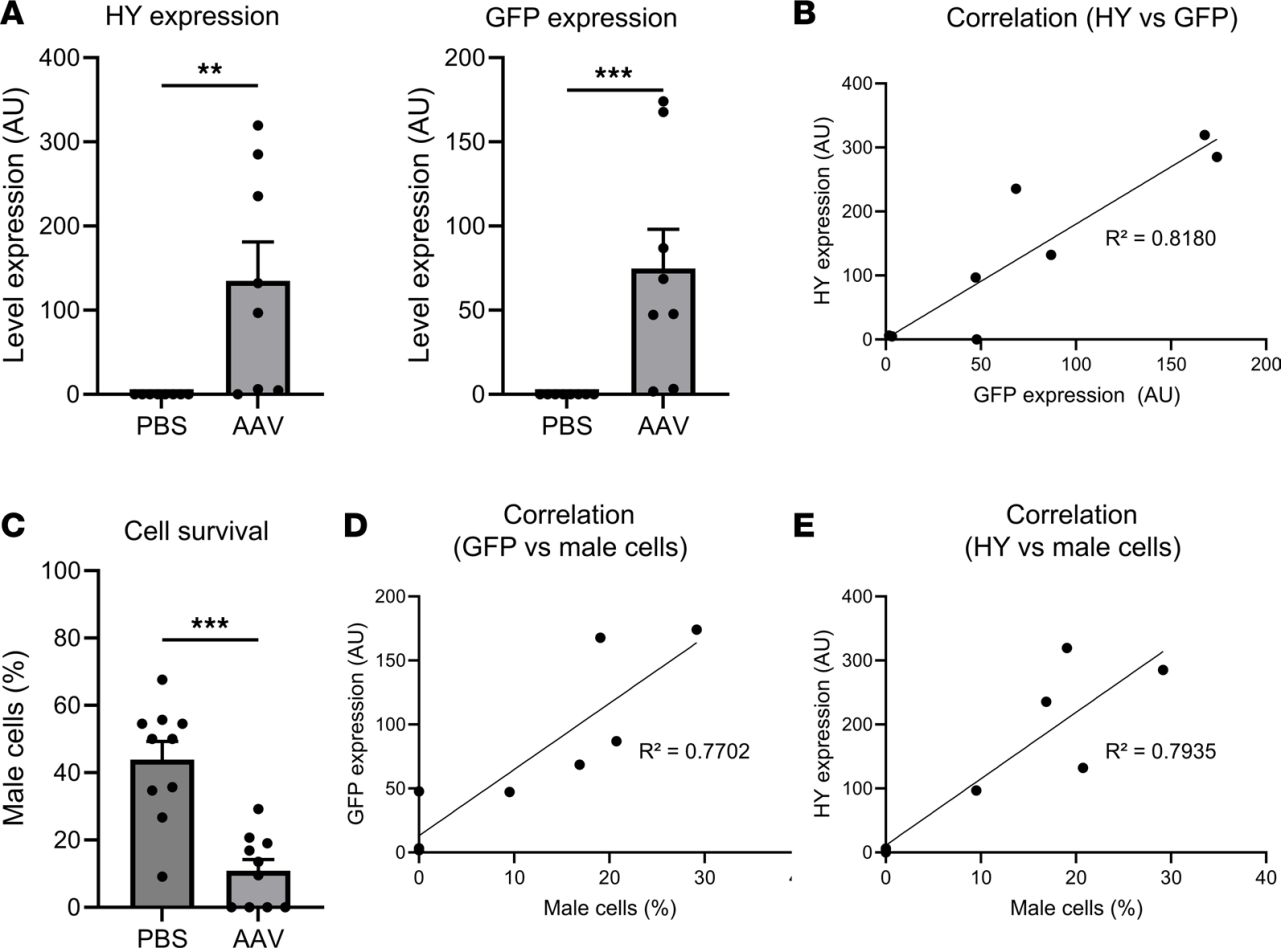
Local transgene expressions in the retina are correlated with cytotoxicity against transgene-positive cells after injection of AAV8-GFP-HY. (**A**) HY and GFP transgene expression levels in female mouse retina 21 days after injection measured by ddPCR. (**B**) Correlation between HY and GFP expressions. (**C**) Activation of cytotoxic cells specific to the HY peptide 21 days after injection evaluated by in vivo cytotoxicity assay. (**D** and **E**) Correlation between the survival of male cells and local transgene expression in AAV-injected mice for GFP (**D**) and HY (**E**). Results obtained from 2 independent experiments (*n* = 8 per group). Bars correspond to mean + SEM. **P < 0.001, ***P < 0.0001 with unpaired Mann-Whitney test. *R*^2^ was calculated via linear regression.

Further, the level of transgene expression was measured to assess the efficacy of AAV8 transduction and its relationship with cytotoxic effects using droplet digital PCR (ddPCR). In AAV8-injected mice, significant expressions of the HY (*P* = 0.0014) and GFP (*P* = 0.0002) transgenes were observed 21 days after injection, and a correlation between the expression levels of both transgenes was identified (*R*^2^ = 0.818) (Figure 5, A and B). To evaluate the clearance ability of specific antigen-expressing cells in these mice, an in vivo cytotoxicity assay was performed using male spleen cells expressing the HY antigen as target cells, which means the more target cells survive, the less cytotoxicity there is. We found a significant reduction (*P* = 0.0004) in the number of male target cells in AAV8-injected mice, indicating the development of HY-specific cytotoxicity (Figure 5C). Direct correlations between target cell survival in the blood and retinal transgene expression (*R*^2^ = 0.7702 for GFP; *R*^2^ = 0.7935 for HY) were demonstrated, suggesting an inverse correlation between cytotoxicity effect and transgene expression in the mice (Figure 5, D and E).

### Local inflammation and correlated expressions between MHC II molecules and Cybb are found in the retina after injection of AAV8-GFP-HY.

**Figure 6:**
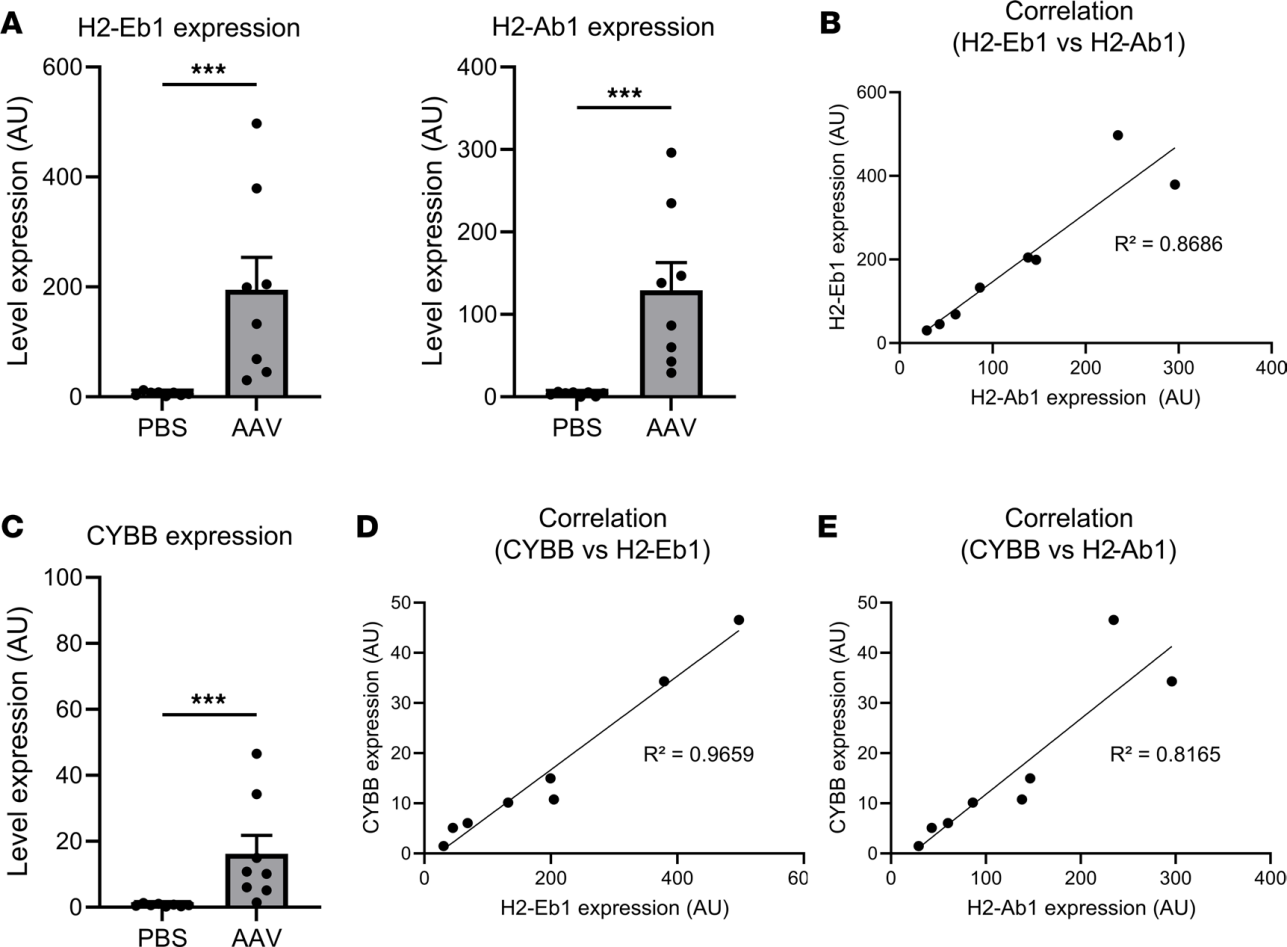
Local inflammation and correlated expressions between MHC II molecules and Cybb are found in the retina after injection of AAV8-GFP-HY. (**A**) Gene expression of H2-Eb1 and H2-Ab1 measured by ddPCR in the mouse retina 21 days after injection. (**B**) Correlation between H2-Eb1 and H2-Ab1 expression levels. (**C**) Gene expression of Cybb measured by ddPCR in the mouse retina 21 days after injection. (**D** and **E**) Correlation between Cybb and H2-Eb1 expression (**D**) and between Cybb and H2-Ab1 expression (**E**). Results obtained from 2 independent experiments (*n* = 8 per group). Bars correspond to mean + SEM. ***P < 0.0001 with unpaired Mann-Whitney test. *R*^2^ was calculated via linear regression.

The direct correlation between target cell survival and retinal transgene expression suggested a potential antigen-presenting cell contribution to present transgene product peptides, associated with local inflammation. This was assessed by evaluation of the transcript expression of MHC II molecules (H2-Eb1 and H2-Ab1) (33) and the type 1 macrophage marker Cybb (34) in the retina, using ddPCR 21 days after the injection. We observed a significant increase in the expression of both MHC II molecules (*P* = 0.0002) 21 days after the subretinal injection (Figure 6A). Since H2-Eb1 and H2-Ab1 are co-dominant molecules, their correlated expressions were confirmed (*R*^2^ = 0.8686) (Figure 6B). Interestingly, the expression of Cybb increased significantly in AAV8-injected mice (*P* = 0.0002) (Figure 6C), and correlations were found between Cybb and MHC II molecule expression (*R*^2^ = 0.9659 for H2-Eb1; *R*^2^ = 0.8165 for H2-Ab1), suggesting a link between inflammatory markers (Figure 6, D and E).

### Diversity in immune responses is noticed in syngeneic mice 21 days after injection of AAV8-GFP-HY.

**Figure 7:**
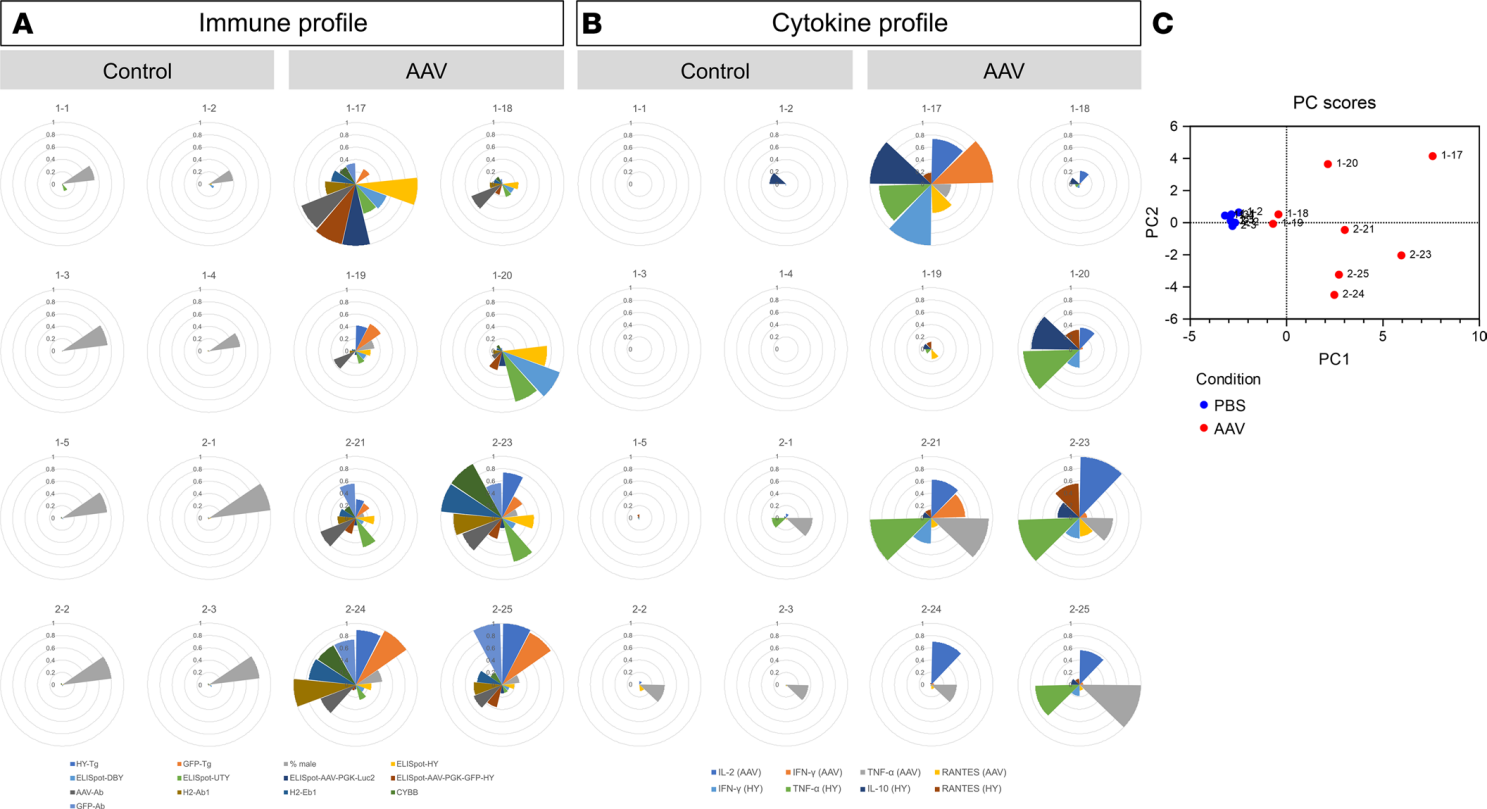
Diversity in immune responses is noticed in syngeneic mice 21 days after injection of AAV8-GFP-HY. (**A**) Immune profiles of each individual mouse showing transgene expression (HY-Tg and GFP-Tg), local inflammation (H2-Eb1, H2-Ab1, and Cybb), and humoral immune response (AAV-Ab and GFP-Ab) and cellular immune response (percent male, ELISpot-HY, ELISpot-DBY, ELISpot-UTY, ELISpot-AAV-PGK-Luc2, and ELISpot-AAV-PGK-GFP-HY) 21 days after injection of PBS (control) and AAV8-GFP-HY (AAV). (**B**) Cytokine profiles of individual mice showing cytokine levels (AAV in vitro stimulation: IL-2, IFNG, TNFA, and RANTES; HY in vitro stimulation: IFNG, TNFA, IL-10, and RANTES) 21 days after injection of PBS (control) and AAV8-GFP-HY (AAV). Each slice of the pie corresponds to one immune parameter, which is normalized against the highest value into percentage for the parameter. Individual animal IDs are shown on top of each radar plot. (**C**) Principal component analysis of PBS-injected mice (blue dots) compared with AAV-injected mice (red dots). Each dot is a data point representing one animal whose individual animal ID is shown beside the dot. The code of mouse is shown above each chart. Results obtained from 2 independent experiments (*n* = 8 per group).

After analysis of all immune parameters typically collected for immunomonitoring in clinical trials, variability in the immune parameters was observed even in this syngeneic model. To figure out the main factors driving this variability, principal component analysis (PCA) was performed, and angle sectors were then used to show the immune profiles and cytokine profiles of each mouse. In angle sectors, all analyzed transgene expression, immune parameters, and significantly regulated cytokines were normalized to their corresponding highest values to provide a more direct visualization. The immune profile included transgene expression (HY-Tg and GFP-Tg), local inflammation (H2-Eb1, H2-Ab1, and Cybb), humoral immune response (AAV8-Ab and GFP-Ab), and cellular immune response (percent male, ELISpot-HY, ELISpot-DBY, ELISpot-UTY, ELISpot-AAV8-PGK-Luc2, and ELISpot-AAV8-PGK-GFP-HY). The cytokine profile was composed of cytokines significantly affected by AAV8 in vitro stimulation (IL-2, IFNγ, TNFα, and RANTES) or HY in vitro stimulation (IFNγ, TNFα, IL-10, and RANTES). No similar immune profiles were found in AAV8-injected mice, suggesting that the immune responses against AAV8 and its transgene product may vary among mice (Figure 7, A and B, and Supplemental Table 3). The PCA results confirmed that AAV8-injected mice could be clearly separated from PBS-injected mice as expected and that the AAV8-injected group exhibited a broad distribution of the individuals that was consistent with immune and cytokine profiles, highlighting the variability of immune parameters after AAV8 subretinal injection (Figure 7C).

### Absence of correlation between immune parameters in syngeneic mice 21 days after AAV8-GFP-HY delivery.

**Figure 8:**
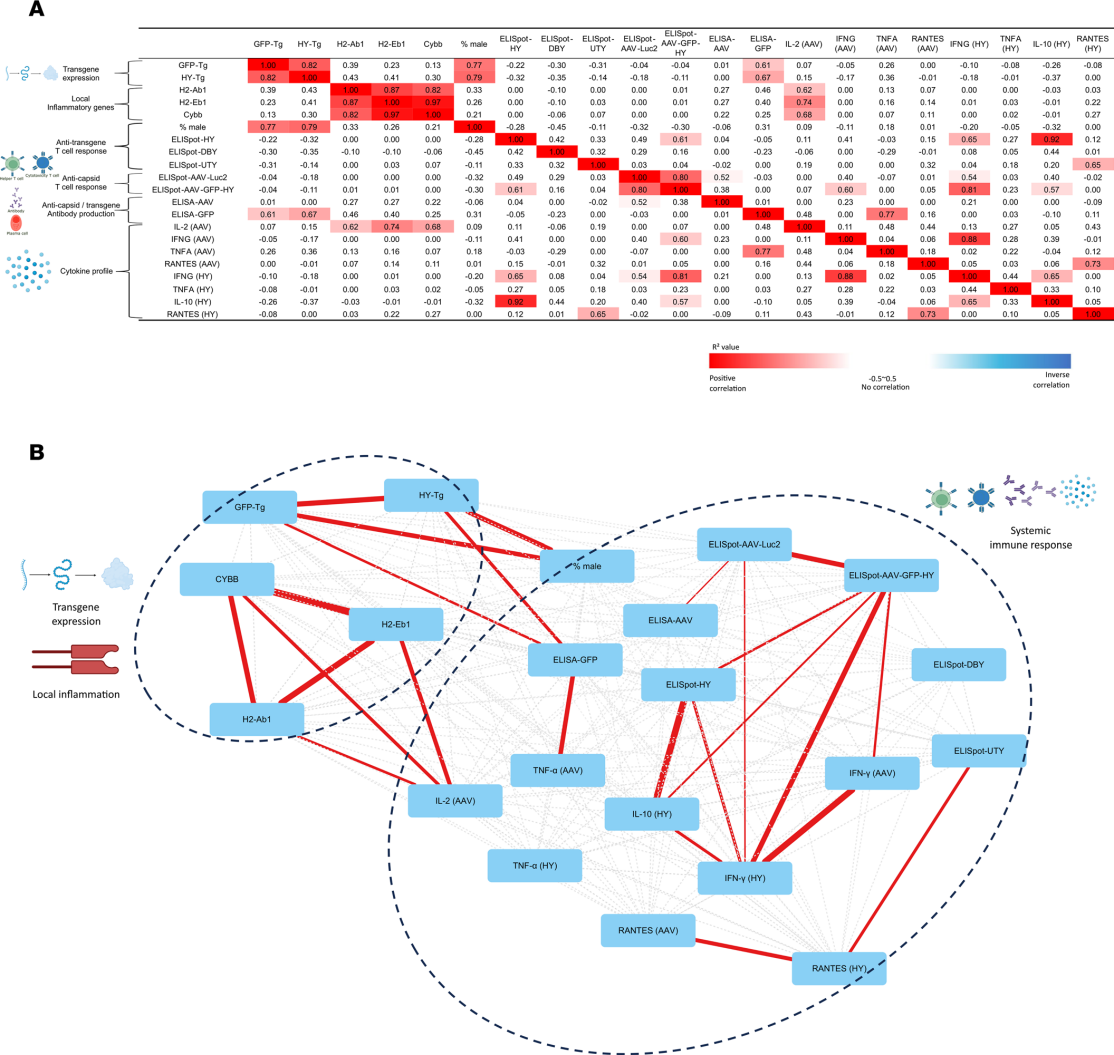
Immune parameters show no correlation in syngeneic mice 21 days after AAV8-GFP-HY delivery. (**A**) Correlation matrix showing the coefficient of determination of each pair of immune parameters tested experimentally. Positive correlations are marked in shades of red with *R*^2^ above 0 and negative correlations in shades of blue with a negative sign before *R*^2^. The intensity of shading corresponds to the strength of correlation. (**B**) Network analysis map generated based on the correlation matrix. The color of the lines connecting 2 parameters (edges) indicates positive (red), negative (blue), or no (gray) correlation, and the thickness of the edges indicates the strength of correlation with thicker lines indicating higher correlation and dotted lines showing no correlation.

To explore relationships among immune parameters, a correlation analysis was conducted. With a correlation matrix, *R*^2^ values for each parameter pair were displayed and were further visualized using a network analysis to illustrate the connections among these parameters. Distinct correlations emerged between certain parameters; for instance, anti–transgene product antibody levels showed a clear correlation with transgene expression, which in turn was linked inversely to male cell death, indicating the cytotoxic effect. However, most parameters displayed no marked correlations, particularly across the 4 primary domains: local transgene expression, local inflammation, systemic immune response, and cytokine profile (Figure 8, A and B). The absence of correlation between immune markers suggests that subretinal AAV8 administration leads to individualized immune responses, with marked variation between subjects. This indicates that each subject may mount a distinct immunological reaction to the treatment.

## Discussion

Gene therapy offers a promising approach for treating genetic diseases, with AAV vectors employed to deliver the therapy (35–37). Nevertheless, immune responses triggered by AAV capsids and transgene products remain a concern, as they are observed both peripherally and locally in preclinical and clinical studies, and they are generally considered as factors that can negatively affect the effectiveness of gene therapy (38–40). We highlight here that immunomonitoring results in clinics vary among patients. The immune response variations observed in patients are usually attributed to the stage of disease, treatment prior to gene therapy, genetics, environment, and lifestyle. Besides, the samples collected from humans are usually not collected on the same day; thus, variability in timing of PBMC isolation and freeze thaw can substantially impact and amplify the variability in the immunomonitoring output (41). However, in our study, despite the use of a syngeneic murine model treated with AAV8 vectors in highly controlled conditions (same sex, same age, kept in the same environment, subjected to the same treatment), a considerable interindividual variability was still observed, similar to the high interindividual variability noticed in both human clinical and NHP data.

Interindividual variability of immune response after intravitreal AAV2 injections was evident in humans and NHPs. None of the individuals, whether human or NHP, who received the same dose and vector injection exhibited identical immune responses. This observation confirms that there is a widespread variability in heterogeneous individuals that has not been well understood, and emphasizes the critical need to identify the factors contributing to this interindividual variability. In order to confirm whether the interindividual variability persists despite the route of administration, transgene, and serotypes, AAV8 vector was applied in murine experiments. Thus, systemic immune response induced by AAV8 subretinal injection was characterized in the syngeneic murine model. HY-GFP was packaged into AAV8, which was previously shown to induce systemic T cell immune responses (12, 31) against transgene products. All the mice developed adaptive systemic immune responses, and, surprisingly, innate immune responses persisted 3 weeks after the injection. In previous studies aiming to explore the factors impacting the AAV-induced immune responses, a dose dependence was repeatedly identified (42, 43), which can also be influenced by serotype (44–46), administration route (47), transgene (10, 48–50), and the sex of the recipient (51). However, in the clinical trial included in our study (Figure 1), there was interindividual variability in immune responses, but this was not dose dependent. In the context of this study, optimization of vector dose and transgenes could help reduce the risk of inducing a potential immune response. Furthermore, developing novel AAV vectors with lower immunogenicity can be another alternative option to evade part of the immune responses directed against the capsid. In addition to directed evolution, capsid shuffling, and rational design, modeling in silico and machine learning are increasingly being used for AAV capsid development. In silico strategies can predict which mutations are likely to enhance AAV functionality and narrow down potential candidates to test in experimental setting (52).

Correlation data analysis revealed an interesting finding: there were very few marked relationships between immune parameters, including local transgene expression, local inflammation, and systemic immune responses. This relative independence of immune factors has not been previously documented. Among the studied parameters, transgene expression level appears to be inversely correlated with the cytotoxicity against transgene-expressing cells. In addition, it also seems in our murine model that the humoral immune response against the transgene is correlated with the transgene product level of expression, but not with cytotoxicity. Further investigations, including other controls and transgene cassettes, should help to clarify whether there is an impact of the transgene type on this absence of correlation between humoral and cellular adaptive immune responses. Interestingly, our results suggested a link between local inflammatory markers in the retina. However, the absence of correlation found with the commonly used clinical immunomonitoring methods, such as ELISA and ELISpot (17, 53, 54), which investigate peripheral adaptive immune responses, suggests that they may not fully capture the complexities of immune reactions in ocular gene therapy patients. Thus, the use of these immunomonitoring techniques may not reflect the actual immune status, and they cannot be used to determine the application of immunosuppression regimens in patients. It would also be informative in further studies in animal models to investigate the potential link between the immune response variability and the percentage of retinal area impacted by the injection, such as with RNA in situ hybridization or immunofluorescence assays on retinal flat-mounts, or a ddPCR assay to quantitatively detect AAV genome or transgene. Systemic cytokine profiles and putative inflammatory biomarkers (55) proved to be poor predictors of immune responses following AAV gene delivery to the eye. However, a more extensive assessment of local cytokine expression patterns could provide additional insights. Besides, patients with retinal diseases express local inflammation cytokines in the retina that have the potential to leak into the periphery, which further complicates the sensitivity of cytokines as biomarkers (56). Therefore, novel immunomonitoring strategies need to be explored to provide a complete and exhaustive view of immunological status, which can further aid in the standardization of the application of immunosuppression strategies in clinics.

Interindividual variability in immune responses has been observed in human clinical trials as well as in NHPs receiving ocular gene therapy. This is expected since even in human monozygotic twins, the immune responses can differ due to T and B cell repertoire (57, 58). However, with our study we demonstrated that even in a syngeneic murine model where most of the factors were kept the same, high interindividual variability in immune responses is observed. Even though factors like the surgery and operation of the experiments cannot be exactly the same in all mice, this kind of artificial variation also exists in human and other animal models (59). Similar interindividual variability of immune response has been reported in vaccine and antitumor research within syngeneic murine models (60–63). The factors and mechanisms impacting the interindividual variability are still not well understood. One study applied vaccinia virus to activate the T cell response in C57BL/6 mice in order to follow CD8^+^ T cell dynamics, and interindividual variability was observed in priming efficiency, effector expansion, and memory cell generation (60). Additionally, another study reported a varied immune response in C57BL/6 mice given concanavalin A, which can induce immune-mediated hepatitis, and measured kinetics of alanine aminotransferase levels (a marker of liver damage), emphasizing that immune responses vary substantially in timing and magnitude, even among genetically identical subjects (61). Besides, a study tracked CD8^+^ cells in a CT26 syngeneic murine tumor model) to assess anti–PD-1 therapy response, and high variability in antitumor responses was observed, indicating a contribution of the rate of CD8^+^ T cell activation to the interindividual variability (62). Another study showed that different intestinal microbiota could impact the immune response in syngeneic mice. Mice that were raised in two different facilities showed differences in CD8^+^ cell priming and accumulation during antitumor therapy (63). In our study, however, all the mice were raised in the same facility, under the same diet and housing conditions, and would be expected to have similar intestinal microbiota. Nonetheless, this can be a factor influencing the variability in human and other animal models and merits consideration. Such studies reinforce the idea that interindividual immune variability is a common phenomenon in immunological contexts beyond gene therapy, and further exploration is needed to elucidate the underlying mechanisms.

An additional aspect to consider when developing and providing gene therapies is the influence of prior AAV exposure on immune outcomes. Presently gene therapies are delivered using AAVs that are quite prevalent, and exposure to AAVs is common among humans and even preclinical models such NHPs. Such exposure does not cause any pathology in humans but elicits an immune response by developing anti-AAV antibodies that are present in the serum. Many studies have reported the prevalence of anti-AAV antibodies in both humans and NHPs that are AAV serotype specific (64).These anti-AAV antibodies can confound gene therapy outcomes, as they can bind to the injected AAVs and neutralize them, or they can further trigger a stronger immune response resulting in inflammation (65, 66). This was a reason for exclusion of patients with preexisting antibodies from early clinical trials (30). But more recent clinical trials often include all patients, especially in the case of the retina when a subretinal mode of delivery is used, as the injected AAVs may remain isolated from circulating anti-AAV antibodies (30, 67). However, in certain disease conditions wherein the blood-retina barrier is compromised, this has to be taken into consideration. A previous study conducted by us on NHPs showed that the preexisting antibodies were a poor indicator of potential immune responses (11).

Animal models provide crucial inputs for gene therapy development and fundamental immunology research. In our study, even though we used a syngeneic murine model to eliminate most internal and external variability factors, an interindividual variability in immune responses was observed. Alternatively, research at the cell and organoid levels, which are used in gene therapy transduction (68), may assist in understanding more about the factors that impact the variability by focusing, for example, on innate immune reactivity. Regardless of the systemic collaboration of immune systems, cell lines and organoid models can still provide insight into immune sensitivity to specific antigens where attempts to use cell culture to predict immunogenicity have been initiated (69, 70).

Our findings underscore the need for individualized patient care strategies, highlighting the value of a personalized medicine approach (71, 72). The understanding and surveillance of individual immune response variations could inform personalized decisions about dosing, treatment selection, duration, and immunosuppressive regimens. The contribution of artificial intelligence (AI) in biology has been expanded widely during the last decade, which allows metadata analysis and predictions. Multiple studies have used AI in cancer immunotherapy, and different AI models have been trained to predict not only various immune signatures but also direct immunotherapy responses (73). Despite the existence of predictive tools of the immune response in specific cells or signaling pathways (74, 75), those have not been adapted to the context of gene therapy, and challenges in predicting immune responses in this field still largely persist. Current limitations in our understanding of immune system complexity may prevent AI from generating accurate predictions.

In humans and complex animal models like NHPs, interindividual differences in immune responses are expected, and attempts are made to minimize their impact on safety and efficacy (immunosuppression strategies) or to resolve symptoms (anti-inflammation strategies), with little or no information on the underlying mechanisms. Our goal with the present study was to identify the most pertinent parameter or a combination of parameters that can be reliably used to predict, follow up, and manage immune responses after therapy. However, even in syngeneic mice receiving the same treatments we observed high variability in immune responses. Few correlations were observed among local transgene expression, local inflammation, and systemic immune responses, revealing the limitation of the current immunomonitoring strategies in ocular gene therapy clinical trials. Our study reinforces two crucial points. First, the current immunomonitoring strategies can hardly be used to infer the ongoing immune response and adapt the immunosuppressive regimens. Efforts should be focused on the understanding of the underlying mechanisms leading to the individual differences observed in the immune response. Second, in the therapeutic context, patients may benefit from personalized therapies designed with consideration of their own unique immune profiles. 

### Limitations of the study

In this study, a limited range of immune parameters were evaluated concerning local inflammation, systemic cytokines, and systemic anti–transgene product and anti-capsid humoral and cellular immune response. These outputs, which correspond to those mostly used in clinical immunomonitoring, may not reflect the complex individual immune responses after AAV subretinal injection. Therefore, a more thorough analysis of local inflammation or the systemic immune response using alternative techniques can strengthen the conclusions regarding immune response variability while also identifying new potential biomarkers and immunomonitoring strategies. Furthermore, all the experiments were performed based on a controlled syngeneic murine model with the same age and sex. Our results should be confirmed in other syngeneic murine models to exclude the potential impact of the model, possibly taking into account additional parameters such as the animals’ age, sex, route of injection, and AAV serotypes.

Further, in this study the humans and NHPs received AAVs by intravitreal injections, whereas the mice were injected subretinally. The time point of sample collections post-injection and the type of immune parameters evaluated were also different. This limits the possibility of making a direct comparison between the 3 models presented, although they point in the same direction — the presence of interindividual differences. However, future evaluations performed under similar conditions and taking into account all the parameters will enable comparison across species and may provide additional insights.

## Methods

### Sex as a biological variable.

In the clinical study, most subjects were male (*n* = 13), and in the NHP study, there were 6 males and 2 females. Sex was not considered as a biological variable because of the limited availability of patients or animals. Since sex has been identified as a factor influencing AAV-induced immune responses (51), mice of the same sex were used to investigate additional contributors to this variability. In the murine study, female mice were selected because HY peptides are male-specific antigens capable of inducing immune responses only in female individuals while allowing the assessment of other factors contributing to the variability observed after AAV administration.

### Clinical data and NHP data analysis.

Immunomonitoring data from the clinical trial (NCT02064569), which included 15 patients with Leber hereditary optic neuropathy carrying the G11778A-ND4 mutation, were analyzed. These patients were divided into 4 dose cohorts (9 × 10^9^, 3 × 10^10^, 9 × 10^10^, and 1.8 × 10^11^ vg per eye), and each cohort received an intravitreal injection. Ocular inflammation, anti-AAV2 TAb, anti-AAV2 NAb, and anti-AAV2 cellular immune response were quantified, and details were described previously (26). In brief, a composite global OIS was determined according to SUN classification. Anti-AAV2 TAbs were determined by ELISA from human serum samples prediluted at 1:50. NAb assay was used to measure anti-AAV2 NAb with HEK293 cells cultured with an rAAV2 expressing luciferase under the control of the cytomegalovirus promoter (8 × 10^7^ vg per well) either alone or with serial fold dilutions of human serum samples. The half-maximal inhibitory concentration was determined using the intercept at 50% of the regression curve and expressed as a dilution factor. PBMCs were isolated, and an IFNγ ELISpot assay was performed to measure cellular immune response against AAV2 antigens. Angle sector diagrams for patients were generated based on the fold change of anti-AAV2 TAb and NAb, maximal OIS, and anti-AAV2 cellular immune response, which had been normalized with the highest value corresponding to each aspect.

Immunomonitoring data from 8 NHPs were analyzed. NHPs received an intravitreal injection of 1 × 10^9^ vg AAV2.7m8 in both eyes. Ocular inflammation and anti-AAV2 TAb and NAb were evaluated, and details were described previously (11). In brief, anti-AAV2 TAbs were determined by ELISA from NHP serum samples prediluted at 1:100. NAb assay for AAV2 was performed using HEK293T cells cultured with an rAAV2 expressing luciferase under the control of the cytomegalovirus promoter (at a multiplicity of infection of 6,400 per well) either alone or with serial fold dilutions of NHP serum samples. The half-maximal inhibitory concentration was determined using the intercept at 50% of the regression curve and expressed as a dilution factor. A SPECTRALIS HRA + OCT system was used to acquire optical coherence tomography (OCT) images. SUN classification was applied to grade the anterior chamber cells. To grade the vitreous cells, the NIH grading system was used. The British Medical Journal (BMJ) grading system was used to grade the posterior uveitis.

### Murine model.

Wild-type 6- to 8-week-old female C57BL/6J mice (H-2^b^) were purchased from Charles River Laboratories. Animals were anesthetized either by intraperitoneal injection of 120 mg/kg ketamine (Virbac) and 6 mg/kg xylazine (Bayer) or by inhalation of isoflurane (Baxter). They were euthanized by cervical elongation.

### AAV vectors.

AAV8-GFP-HY and AAV8-PGK-Luc2 vectors were produced by Genethon in Evry (France) using the tritransfection technique in 293T cells cultured in roller bottles (76). Transgenes were under the ubiquitous phosphoglycerate kinase (PGK) promoter. HY is a male antigen that is immunogenic in female mice. AAV vectors were purified by cesium chloride gradient centrifugation, and vector titers were determined by qPCR. Endotoxin levels were below 6 EU/mL.

### Peptides.

The DEAD box polypeptide 3Y-linked and ubiquitously transcribed tetratricopeptide repeat gene, Y-linked (UTY), peptides, NAGFNSNRANSSRSS and WMHHNMDLI, respectively, were synthesized by Genepep and shown to be more than 95% pure. UTY and DBY are immunodominant peptides of the HY antigen, restricted to MHC I and MHC II, respectively.

### Injections in mice.

Injections were performed as described previously (12). Briefly, the right eye was protruded under microscopic visualization, and the sclera was perforated with a 27G beveled needle. A blunt 32G needle set on a 10 μL Hamilton syringe was inserted in the hole, and the same volume (2 μL) of PBS, or AAV (5 × 10 vg), was injected into the subretinal space via the vitreous. The quality of the injection was verified by checking the detachment of the retina and the absence of reflux outside the eye.

### Cell extraction from murine spleen.

After euthanasia, cells were extracted from the spleen as described previously (31). Briefly, spleens were removed and crushed with a syringe plunger on a 70 μm filter in 2 mL of RPMI medium. Red cells were lysed by addition of ACK buffer (8.29 g/L NH_4_Cl, 0.037 g/L EDTA, and 1 g/L KHCO_3_) (MilliporeSigma) for 1 minute. Lysis was stopped by addition of complete RPMI medium (10% FBS, 1% penicillin/streptomycin, 1% glutamine, and 50 μM β-mercaptoethanol). After centrifugation, cells were counted, and the concentration was adjusted in complete RPMI medium.

### Murine IFNγ ELISpot assay.

IFNγ ELISpot assay was performed as described previously (31). IFNγ enzyme-linked immunospot (ELISpot) plates (MAHAS4510, Millipore) were coated with anti-IFNγ antibody (eBioscience) overnight at 4°C. Stimulation medium (complete RPMI), AAV (1 × 10^11^ vg/mL), UTY (2 μg/mL), DBY (2 μg/mL), UTY plus DBY (2 μg/mL), or concanavalin A (MilliporeSigma) (5 μg/mL) was plated, and 5 × 10^5^ spleen cells per well were added. After 24 hours of culture at 37°C, plates were washed, and the secretion of IFNγ was revealed with a biotinylated anti-IFNγ antibody, streptavidin–alkaline phosphatase (Roche Diagnostics), and BCIP/NBT (Mabtech). Spots were counted with an AID ELISpot Reader system ILF05 and the AID ELISpot Reader v6.0 software. Results are expressed in index where IFNγ secretion of the positive control was set to 100, based on the positive control for anti-HY immune response, in order to compile and to compare results from different experiments.

### Cytokine titration by multiplex cytometric bead array.

Cytokine titration by multiplex cytometric bead array was performed as described previously (31). Stimulation medium (complete RPMI), AAV (1 × 10^11^ vg/mL), UTY (2 μg/mL), DBY (2 μg/mL), UTY plus DBY (2 μg/mL), or concanavalin A (MilliporeSigma) (5 μg/mL) was plated, and 1 × 10^6^ spleen cells per well were added. After 36 hours of culture at 37°C, supernatants from triplicates were pooled and frozen at –80°C until the titration. Cytometric bead arrays were performed with BD Biosciences flex kits (IL-1β, IL-2, IL-4, IL-6, IL-10, IL-13, IL-17, RANTES, IFNγ, GM-CSF, TNFα, and MCP-1). Briefly, capturing bead populations with distinct fluorescence intensities and coated with cytokine-specific capture antibodies were mixed together. Next, 25 μL of the bead mix was distributed, and 25 μL of each sample (supernatants) was added. After 1 hour of incubation at room temperature, cytokine-specific PE-antibodies were mixed, and 25 μL of this mix was added. After 1 hour of incubation at room temperature, beads were washed with 1 mL of wash buffer, and data were acquired with an LSRII flow cytometer (BD Biosciences). FCAP software (BD Biosciences) was used for the analysis.

Radar diagrams represent the percentage of cytokine secretion in the different groups based on the maximum of cytokine secretion and were generated with Excel software. The 100% radar scale fits the maximum value of cytokine secretion, and the black area values correspond to the means of these cytokine secretions.

### In vivo cytotoxicity assay.

IVC assay was performed as previously described (12).

Spleen cells from CD45.1^+^CD45.2^–^ male (expressing HY antigen) and CD45.1^–^CD45.2^+^ female (not expressing HY antigen) C57BL/6 wild-type mice were harvested as described above and stained with a CellTrace Violet (CTV) cell proliferation kit (Molecular Probes) in PBS at different concentrations, 2 μM for male and 20 μM for female cells, according to the protocol of the kit. CTV staining level allows separate tracking of male and female transferred cells. A mixture of 3 × 10^6^ male cells (CTV^low^) and 3 × 10^6^ female cells (CTV^high^) in 200 μL was injected intravenously in the experimental female C57BL/6 mice (CD45.1^–^CD45.2^+^ and not expressing HY antigen) at day 17 of the protocol. CD45.1^–^CD45.2^+^ CTV^high^ female cells are used as a control of cell survival, as they are not targeted by anti-HY immune responses. Three days after injection, blood was harvested, red blood cells were lysed by addition of ACK buffer and washed in 1× PBS, and leukocytes were stained for flow cytometry with an anti-CD45.1–PE antibody (BD Biosciences) to analyze male cell survival in vivo (Pharmingen, BD Biosciences). Data were acquired on a CytoFLEX LX flow cytometer (Beckman Coulter) and analyzed with the CytExpert software (Beckman Coulter).

### Enzyme-linked immunosorbent assay.

Ninety-six-well Maxisorp plates (Thermo Fisher Scientific) were coated with full AAV8 capsid (5 × 10^8^ vg/well) or GFP (0.5 μg/well; Chromotek) diluted with coating buffer (0.84% NaHCO_3_, 0.356% Na_2_CO_3_, pH 9.5) (MilliporeSigma) overnight at 4°C. The wells were emptied and washed with blocking buffer (1× PBS/6% milk) before incubation of the plate with blocking buffer at room temperature for 2 hours. Serial dilutions of primary antibodies (anti-AAV8 antibody, Humimmu; anti-GFP antibody, Abcam) or serum were prepared during the incubation. Primary antibodies were diluted with dilution buffer (1× PBS/1% BSA) to ensure a gradient on the plate (100 ng, 50 ng, 25 ng, 12.5 ng, 6.25 ng, 3.2 ng, 1.56 ng, 0.781 ng, 0.391 ng, 0.195 ng, 0.0975 ng for AAV8; and 25 ng, 12.5 ng, 6.25 ng, 3.2 ng, 1.56 ng, 0.781 ng, 0.391 ng, 0.195 ng for GFP) to calculate the standard curve for the experiment. Murine sera were diluted to 1:1,000 with dilution buffer for AAV ELISA and 1:2,500 for GFP ELISA. After the incubation, primary antibody dilution and serum dilution were added in the wells, and the plate was incubated for 1 hour in the incubator at 37°C after washing 3 times with washing buffer (1× PBS/0.05% Tween 20). Dilution of secondary antibodies (Goat Anti-Mouse IgG, SouthernBiotech) at 1:4,000 was prepared with dilution buffer. Secondary antibodies were added into each well after washing 3 times, and the plate was incubated for 1 hour in a 37°C incubator. The TMB reagent (BD Biosciences) and stop solution (1 M sulfuric acid) were placed at room temperature 30 minutes before the end of the previous incubation. When the last incubation ended, the plate was washed 3 times with washing buffer. TMB reagent was added, and a blue color appeared. Stop solution was added to stop the reaction after 10 minutes. The plate was read at 450 nm to get the optical density of each sample.

### RNA extraction from mouse model retinas and reverse transcription.

Total RNA was isolated using an RNeasy kit (74106, QIAGEN). The procedure was performed according to the manufacturer’s specification. The purification included a DNase treatment using the RNase-Free DNase Set (QIAGEN). The yield and purity of the RNA were measured with a NanoDrop 8000 spectrometer. A Verso cDNA Synthesis Kit (Thermo Fisher Scientific) was applied for reverse transcription following the protocol provided.

### Droplet digital PCR.

For each gene, a mix of primer and probe was prepared with 18 μL of forward primers, 18 μL of reverse primers, 5 μL of probe (initial concentration of 100 mM; Eurofins Genomics), and 59 μL of H_2_O MilliQ. Eleven microliters of droplet digital PCR (ddPCR) Supermix for probes (no dUTP) (Bio-Rad), 1 μL from the primers/probes mix prepared previously (the ratio of housekeeping gene and target gene was 1:1), 6 μL of water, and 2 μL of the cDNA sample from mice (diluted with water to obtain quantities of either 5 ng or 2.5 ng in the wells) were added in one well. The droplets were generated by an Automated Droplet Generator (Bio-Rad). A PCR was run by the C1000 Touch Thermal Cycler (Bio-Rad), with the program as 10 minutes at 95°C, repetition of 30 seconds at 94°C and 1 minute at 58.1°C 40 times, then 10 minutes at 98°C to finalize at 12°C infinite hold. The results were obtained by a QX 200 Droplet Reader (Bio-Rad) with the software QXManager for the analysis. Copy numbers of the genes were obtained. Copy number of gene of interest was normalized with copy number of housekeeping gene, and the ratio was multiplied by 100.

### Statistics.

Statistical analyses were performed with GraphPad Prism v10.0. Mann-Whitney tests, correlation matrix, and principal component analysis were performed. *P* values of less than 0.05 were considered significant. Radar diagrams and angle sector diagrams made by Excel software represent the percentage of measured value in the different groups normalized with the maximal value in the corresponding aspect. Network analysis was performed with Cytoscape v3.10.2 (https://cytoscape.org/). The computation of half-maximal inhibitory concentration of NAb (IC_50_) was performed using R. The schematics were created in BioRender (Dalkara D, 2026, https://BioRender.com/r6f6pqo).

### Study approval.

The clinical study received approval of the French Ethics Committee and adhered to the tenets of the Declaration of Helsinki; it was registered on ClinicalTrials.gov (NCT02064569) (77).

NHP experiments and procedures were ethically approved by the French Ministère de l’Education, de l’Enseignement Supérieur et de la Recherche, and were carried out according to institutional guidelines in adherence with the NIH *Guide for the Care and Use of Laboratory Animals* (National Academies Press, 2011) as well as Directive 2010/63/EU of the European Parliament (78).

All mice were housed, cared for, and handled in accordance with European Union guidelines and with the approval of the local research ethics committee (CEEA-51 Ethics Committee in Animal Experimentation, Evry, France; authorization 2015102117539948).

### Data availability.

This study includes no data deposited in external repositories, and values for all data points in graphs are reported in the Supporting Data Values file. Requests for further information and for resources and reagents should be directed to and will be fulfilled by the corresponding author. Any additional information required to reanalyze the data reported in this paper is available from the corresponding author upon request. The Primer list and Antibody list are provided in the supplemental materials.

## Author contributions

SF, DA, and DR conceptualized the study. DR, GAC, JV, EC, and AP developed methodology. SF, DA, and DR performed investigation. DA and DR wrote the original draft of the manuscript. CVC, JP, DR, DA, AG, DD, HS, and SF reviewed and edited the manuscript. AG, DD, and GR acquired funding. CVC provided resources. DA and SF supervised the study.

## Funding support

- Fondation de France (Berthe Fouassier).
- LabEx LIFESENSES (ANR-10-LABX-65).
- IHU FOReSIGHT (ANR-18-IAHU-01).
- Conseil Régional d’Ile-de-France (DIM C-BRAINS).
- China Scholarship Council (202108070132).

## Supplementary Material

Supplemental data

Unedited blot and gel images

Supporting data values

## Version 1 02/18/2026

In-Press Preview

## Version 2 04/08/2026

Electronic publication
