# Supplementary material for: Interindividual variability in immune response to AAV ocular gene delivery across species impedes immunomonitoring
Source: JCI Insight. 2026 Feb 17;11(7):e199587. doi: 10.1172/jci.insight.199587 (PMC13134722; doi:10.1172/jci.insight.199587)
Supplement: Supplemental data [file jciinsight-11-199587-s306.pdf]

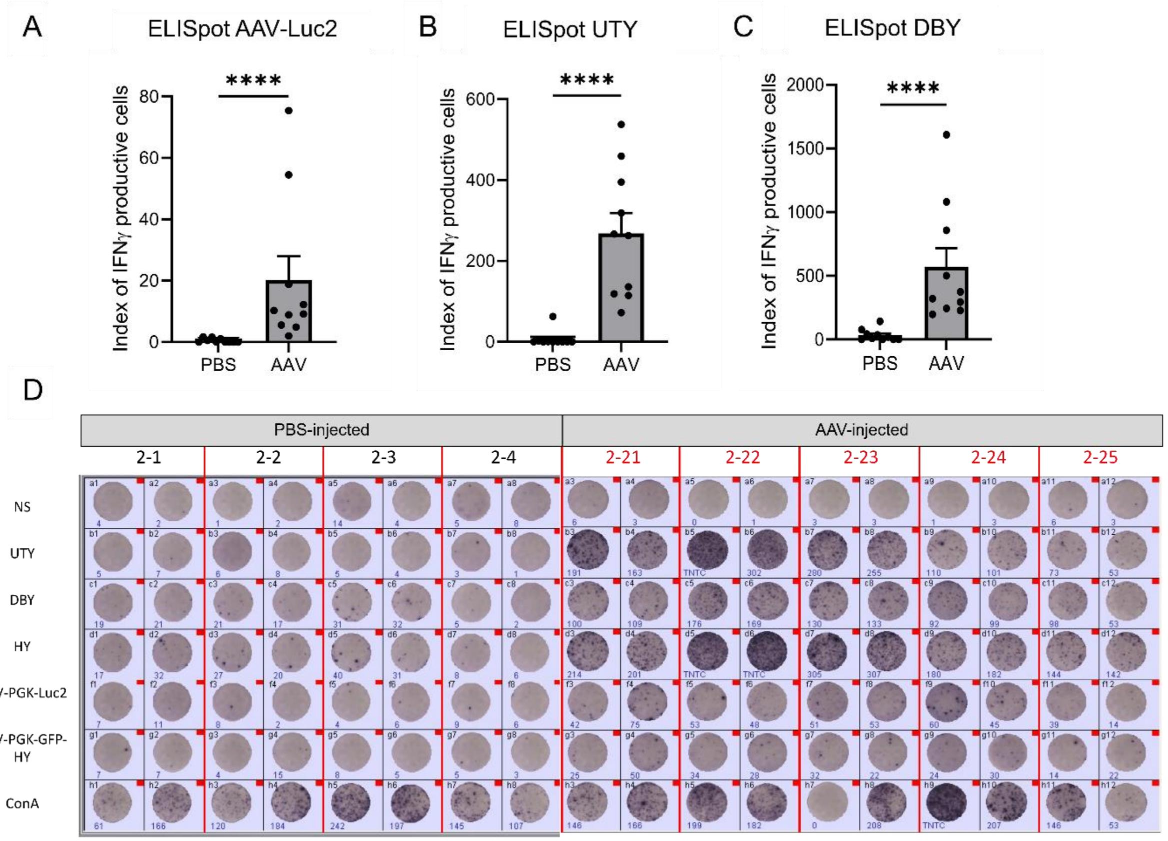

Supplementary Figure 1. CD4^{+} T cell and CD8^{+} T cell activation by peptides.
A T cell activation specific to AAV capsid measured by ELISpot assay.
B CD8^{+} T cell activation specific to UTY peptides.
C CD4^{+} T cell activation specific to DBY peptides.
D Representative ELISPOT images.
ConA: Concanavalin A. Data information: Results obtained from 2 independent experiments (n=10 per group). Bars correspond to mean + SEM. *P < 0.05, **P < 0.01, ***P < 0.001, and ****P < 0.0001 with unpaired Mann-Whitney test.

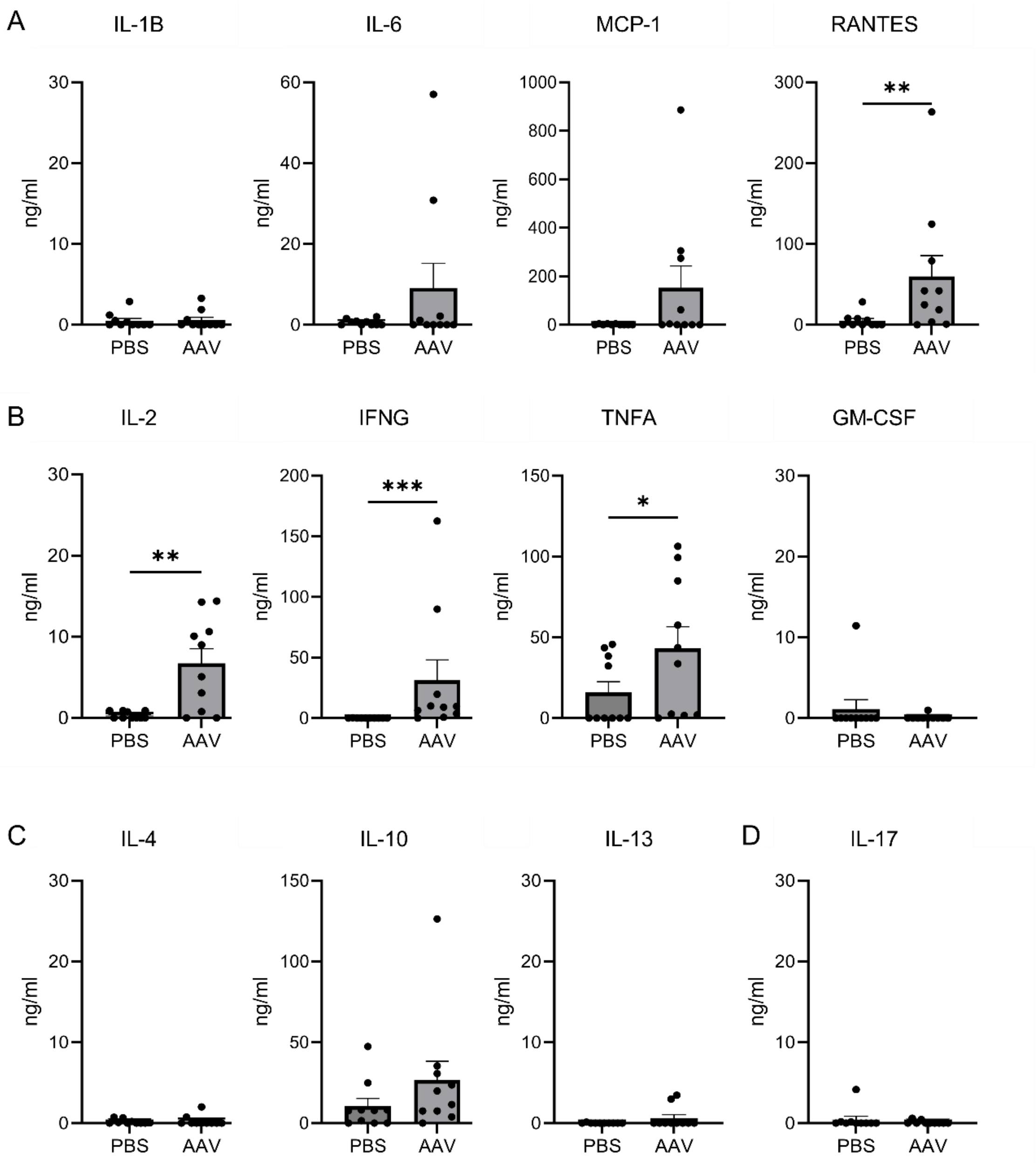

Supplementary Figure 2. Cytokine secretion in mouse spleen cells 21d post-injection (PI) of AAV8-GFP-HY stimulated with AAV *in vitro*.

A-D Expression of cytokines participating in **(A)** Inflammation and cellular migration (IL-1b, IL-6, MCP-1, RANTES), **(B)** Th1 function (IL-2, IFNG, TNFA, GM-CSF), **(C)** Th2 function (IL-4, IL-10, IL-13) and **(D)** Th17 function (IL-17).

Data information: Results obtained from 2 independent experiments (n=10 per group). Bars correspond to mean + SEM. *P < 0.05, **P < 0.01, ***P < 0.001, and ****P < 0.0001 with unpaired Mann-Whitney test.

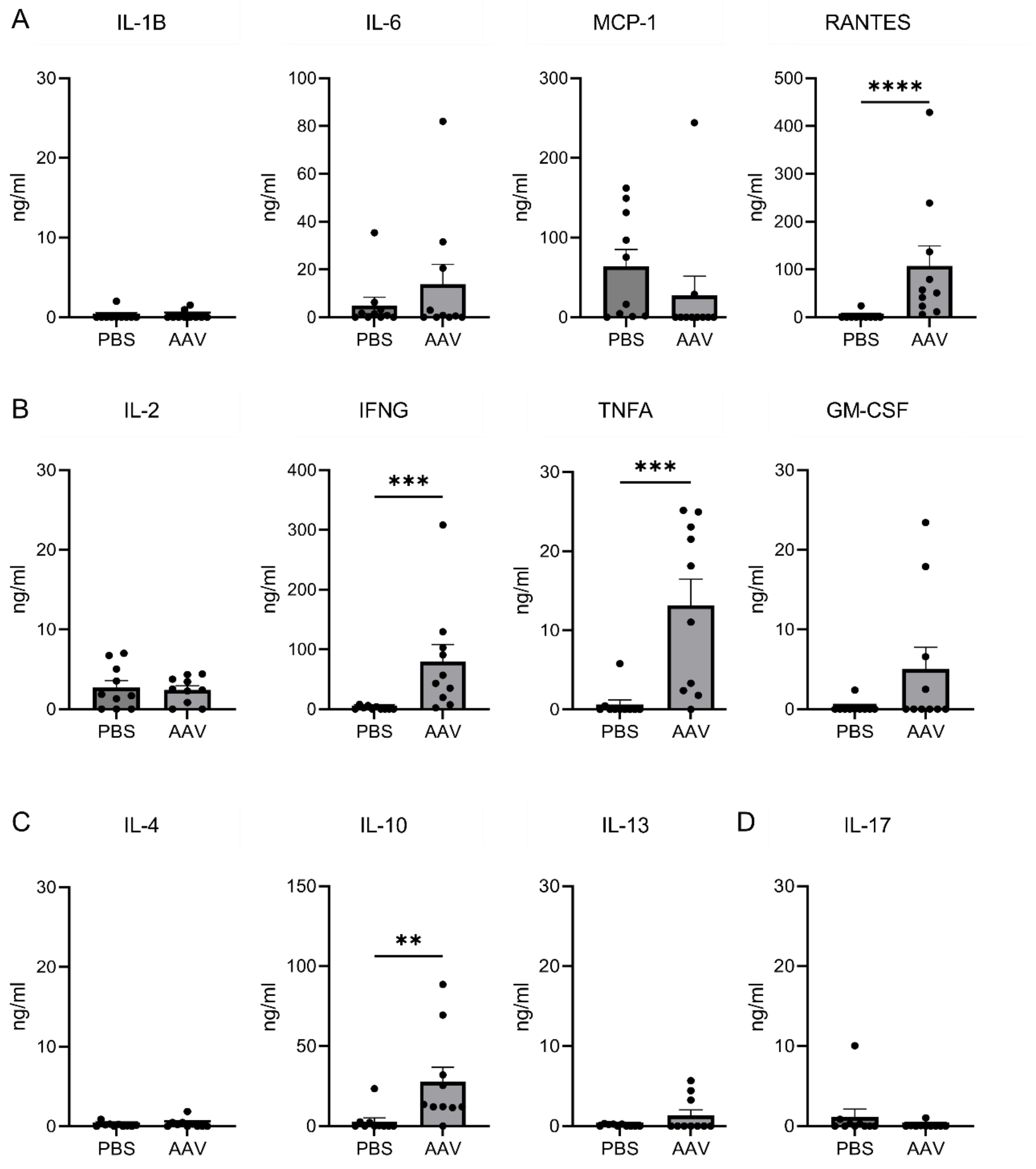

Supplementary Figure 3. Cytokine secretion in mouse spleen cells 21d post-injection (PI) of AAV8-GFP-HY stimulated with HY peptides (pHY) *in vitro*.

A-D Expression of cytokines participating in **(A)** Inflammation and cellular migration (IL-1b, IL-6, MCP-1, RANTES), **(B)** Th1 function (IL-2, IFNG, TNFA, GM-CSF), **(C)** Th2 function (IL-4, IL-10, IL-13) and **(D)** Th17 function (IL-17).

Data information: Results obtained from 2 independent experiments (n=10 per group). Bars correspond to mean + SEM. $^{*}P < 0.05$, $^{**}P < 0.01$, $^{***}P < 0.001$, and $^{****}P < 0.0001$ with unpaired Mann-Whitney test.

Supplementary Table 1. Characteristics of individual immune responses in patients

Dose Level, Patient No.	Maximum, OIS	IgG ($\times 1^{-3}$ AU/mL)		NAb (IC_{50})		Cellular Response
		Baseline	Maximum Value	Baseline	Maximum Value	
9E9 vg						
001	0	7115	7565	1 440	3050	Negative
003	0.5	3520	14895	520	3650	Negative
005	0	1903	5355	360	3400	Positive
3E10 vg						
006	1	155	10665	23	2200	Negative
007	1.5	33	336	0	40	Negative
008	0.5	536	2585	48	488	Negative
9E10 vg						
009	5	2110	14240	420	3200	Negative
011	1.5	13	1148	0	120	Negative
012	1	32	177	0	60	Negative
017	0.5	918	1215	228	396	Negative
018	9.5	10295	67240	2850	40 000	Positive
019	1.5	26	78	0	24	Negative
1.8E11 vg						
013	1.5	46	760	0	68	Negative
014	1	4952	18010	630	8400	Negative
015	0.5	155	1229	0	220	Negative

AU, arbitrary unit; IC_{50}, half maximal inhibitory concentration;
NAb, neutralizing antibody; OI, ocular inflammation; OIS, ocular inflammation score; vg, viral genome.

Supplementary Table 2. Characteristics of individual immune responses in NHPs

No.	Eye	Anti-AAV2 TAb (%)		Anti-AAV2 NAb (IC_{50})		Inflammation at Month 1			
		T0	T2	T0	T2	ACC	ACF	VH	VC
NHP1	LE	100	453.86	23.64	443.83	3	1	0	1
	RE					1	0	0	1
NHP2	LE	100	307.03	3.35	487.05	0.5	0	0	1
	RE					0.5	0	0	1
NHP3	LE	100	537.08	3.56	159.55	0	0	0	1
	RE					0.5	0	0	1
NHP4	LE	100	408.2	1.57	309.11	1	0	0	1
	RE					1	0	0	1
NHP5	LE	100	142.86	3.16	17.7	1	0	0	1
	RE					1	0	0	1
NHP6	LE	100	383.36	7.94	247.3	0.5	0	0	1
	RE					1	0	0	1
NHP7	LE	100	366.88	8.74	432.64	0.5	0	0	1
	RE					0.5	0	0	1
NHP8	LE	100	230	100	181.75	1	0	0	1
	RE					1	0	0	1

TAb, total antibody; NAb, neeutralizing antibody; IC_{50}, half maximal inhibitory concentration;
ACC, anterior chamber cell; ACF, anterior chamber flare; VH, vitreous haze; VC, vitreous cell; LE, left eye; RE, right eye.

Supplementary Table 3. Characteristics of individual immune responses in mice

Mouse No.	Local transgene and inflammation					Systemic cellular immune response						Humoral immune response		Cytokine profile								
	ddPCR (Arbitraty Units)					ELISpot (Index of IFNG productive cells)					IVC (%)	ELISA (ng/ml)		CBA, AAV *in vitro* stimulation (ng/ml)				CBA, HY *in vitro* stimulation (ng/ml)				
	GFP	HY	H2-Ab1	H2-Eb1	Cybb	UTY	DBY	HY	AAV-GFP-HY	AAV-Luc2	% male	Anti-AAV TAb	Anti-GFP TAb	IL-2 (AAV)	IFNG (AAV)	TNFA (AAV)	RANTES (AAV)	IL-2 (HY)	IFNG (HY)	TNFA (HY)	IL-10 (HY)	RANTES (HY)
1-1	0.01	0.00	0.36	1.04	0.19	62.96	8.93	8.38	0.00	1.64	35.71	1.66	0.00	0.00	0.00	0.00	0.00	0.00	0.00	0.00	0.00	0.00
1-2	0.04	0.00	2.72	3.07	0.48	0.00	142.86	36.31	0.41	0.00	26.67	1.95	0.00	0.00	0.00	0.00	0.00	1.85	6.21	0.00	23.43	0.00
1-3	0.02	0.00	0.00	3.17	0.26	0.00	0.00	0.00	0.00	0.00	50.00	1.66	0.00	0.00	0.00	0.00	0.00	0.00	0.00	0.00	0.00	0.00
1-4	0.03	0.01	5.64	5.32	0.60	0.00	0.00	2.79	0.41	0.00	34.69	2.25	0.00	0.00	0.00	0.00	0.00	0.00	0.00	0.00	0.00	0.00
1-5	0.07	0.00	4.47	7.75	0.78	0.00	35.71	0.00	0.00	0.00	50.00	2.83	0.00	0.00	0.00	0.00	0.00	6.74	8.06	0.45	2.31	23.79
2-1	0.00	0.00	6.30	12.11	0.66	0.70	18.68	18.96	1.39	1.11	67.59	22.64	0.00	0.92	0.00	45.69	8.10	7.00	0.00	5.80	2.64	0.00
2-2	0.00	0.00	4.58	7.86	1.30	2.11	42.02	18.96	0.56	1.67	54.55	25.33	0.00	0.75	0.00	43.52	28.28	5.04	2.03	0.00	0.33	0.00
2-3	0.00	0.00	5.46	8.27	1.08	0.00	77.82	23.24	1.11	0.56	55.68	22.52	0.00	0.00	0.39	38.48	5.88	3.54	4.30	0.00	0.00	0.00
1-17	47.83	0.00	146.78	199.45	14.99	266.67	857.14	865.92	71.31	75.41	0.00	7866.66	267205.17	10.63	162.53	33.69	124.50	3.77	308.17	21.50	88.62	79.45
1-18	1.72	6.03	60.10	68.57	6.06	118.52	321.43	226.26	13.11	2.05	0.00	4473.36	58669.90	3.08	3.71	2.06	3.71	2.41	19.65	1.79	13.62	5.90
1-19	86.96	132.01	29.15	30.37	1.49	114.81	294.64	212.29	0.41	4.92	20.74	3166.20	19897.68	0.00	0.00	2.61	42.08	0.00	2.33	2.39	11.59	50.94
1-20	3.28	4.89	42.95	45.05	5.11	459.26	1607.14	631.28	22.95	18.85	0.00	1478.52	36187.40	5.10	9.01	1.70	0.00	4.35	90.91	23.07	69.44	137.00
2-21	47.30	96.70	86.38	132.65	10.15	262.92	227.24	263.00	18.64	9.18	9.52	5077.02	439856.61	9.00	89.96	99.32	42.01	0.00	129.36	24.97	12.23	57.40
2-23	68.53	235.40	234.72	497.38	46.56	395.08	373.54	444.04	24.77	12.24	16.88	5780.30	444835.37	14.28	19.68	57.61	79.23	2.54	102.78	25.17	32.01	238.93
2-24	174.13	285.04	296.05	379.32	34.32	136.38	244.36	222.63	6.68	5.57	29.17	5030.14	580257.57	10.10	6.37	43.72	18.61	2.30	7.34	0.00	0.00	11.48
2-25	167.71	319.50	138.01	204.76	10.77	72.41	197.67	169.42	26.71	10.30	19.05	4127.59	788037.71	8.08	9.73	106.33	24.88	4.42	56.76	18.15	12.12	41.35

IVC, in vivo cytotoxicity; CBA, cytometric bead array.

Primer list

GAPDH forward primer	Eurofins genomics	CATGGCCTTCCGTGTTCCTA
GAPDH reverse primer	Eurofins genomics	GCGGCACGTCAGATCCA
GAPDH probe (HEX)	Eurofins genomics	CCCCAATGTGTCCGTC
eGFP forward primer	Eurofins genomics	ACGTCTATATCATGGCCGAC
eGFP reverse primer	Eurofins genomics	GTGCTCAGGTAGTGGTTGTC
eGFP probe (FAM)	Eurofins genomics	ACGGCCCCGTGCTGCTGCCC
HY forward primer	Eurofins genomics	CCAATAGCAGCCGAAGTAGT
HY reverse primer	Eurofins genomics	GGTGCATCCAACCTAACTG
HY probe (FAM)	Eurofins genomics	TGGTGGAGGTGGCTATGGAGG
H2-Eb1 forward primer	Eurofins genomics	CTGGTCCGAAATGGAGACTG
H2-Eb1 reverse primer	Eurofins genomics	TGTGCTTTCCACTCGACC
H2-Eb1 probe (FAM)	Eurofins genomics	ACCTGCCAGGTGGAGCATCCCAGCC
H2-Ab1 forward primer	Eurofins genomics	TGTGCAGACACAACTACGAG
H2-Ab1 reverse primer	Eurofins genomics	GACATTGGGCTGTTCAAGC
H2-Ab1 probe (FAM)	Eurofins genomics	ACCCACACCTCCCTGCGGCG
CYBB forward primer	Eurofins genomics	TCGAAAACTCCTTGGGTCAG
CYBB reverse primer	Eurofins genomics	TCTTCGAATCCTTGTCGAGC
CYBB probe (FAM)	Eurofins genomics	CGGGCACCTGCAGCCTGCCTGAATT

Antibody list

IFNγ Monoclonal Antibody	eBiosciences	Cat#14-7318-81; RRID:AB_468475
PE Mouse Anti-Mouse CD45.1	BD Biosciences	Cat#553776; RRID:AB_395044
Anti-AAV8 antibody	Humimmu	Cat#A20016-100ug; RRID:AB_3675841
Anti-GFP antibody	Abcam	Cat#ab1218; RRID:AB_298911
Goat Anti-Mouse IgG, Human ads-HRP	Southern Biotech	Cat#1030-05; RRID:AB_2619742
