# Supplementary figures and images for: Interindividual variability in immune response to AAV ocular gene delivery across species impedes immunomonitoring
Source: JCI Insight. 2026 Feb 17;11(7):e199587. doi: 10.1172/jci.insight.199587 (PMC13134722; doi:10.1172/jci.insight.199587)

Full unedited blot image for Supplementary Figure 1D.

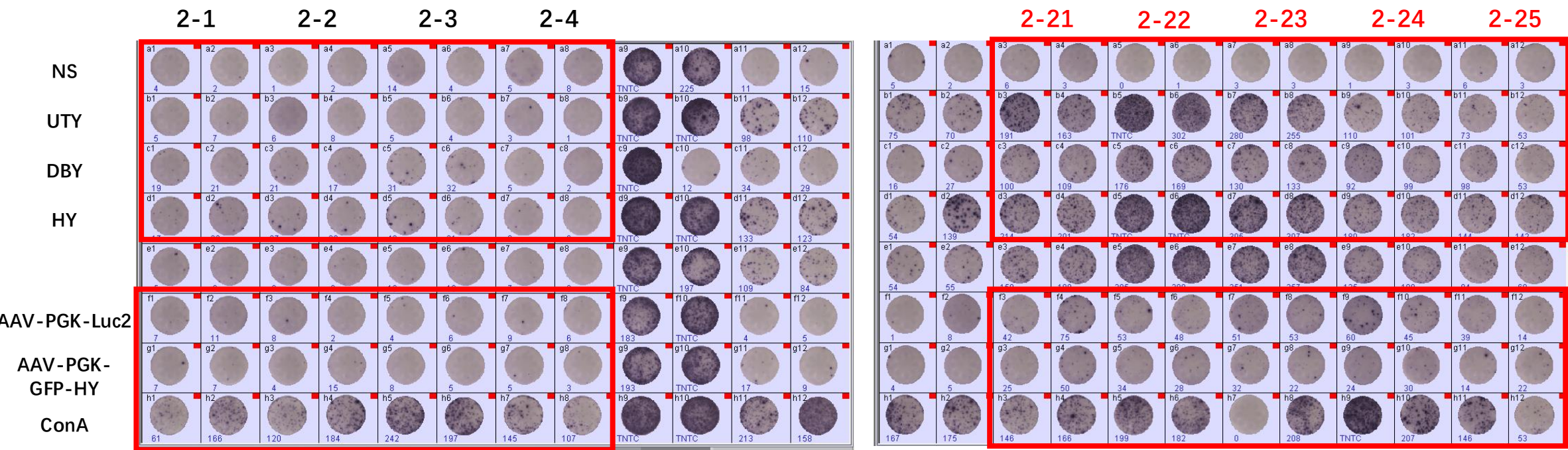

Supplement: Unedited blot and gel images [file jciinsight-11-199587-s307.pdf]
